# Exploring the link between pediatric headaches and environmental noise exposure

**DOI:** 10.1186/s12887-023-04490-4

**Published:** 2024-02-02

**Authors:** Sunho Lee, Kyung-Ran Kim, Wanhyung Lee

**Affiliations:** 1 Department of Pediatrics, CHA Ilsan Medical Center, CHA University, Goyang, Republic of Korea; 2 Department of Pediatrics, Gyeongsang National University Changwon Hospital, Changwon, Republic of Korea; 3 Department of Preventive Medicine, College of Medicine, Chung-Ang University, Seoul, Republic of Korea

**Keywords:** Children, Adolescent, Headache, Noise, Exposure, Stress

## Abstract

**Background:**

Headaches are the most common neurologic symptoms in the pediatric population. Most primary headache in children and adolescents focuses on associated factors, including noise. Auditory discomfort is related to recognizing the pain. We aimed to analyze the headache profile of pediatric populations and the connection between noise exposure and head pain in children and adolescents.

**Methods:**

We reviewed retrospectively medical records of the pediatric population with headaches in Gyeongsang National University Changwon Hospital from January 2022 to April 2023. Personal headache profiling from self-questionnaires and environmental noise data from the National Noise Information System (NNIS) were used to analyze each variable, and chi-square tests and linear regression models by SAS were used to analyze the statistical correlation.

**Results:**

Of the 224 participants, 125 were clinically diagnosed with headaches. Of the 104 pubertal subjects, 56.7% were diagnosed with headaches, compared to 60% in the prepubertal group. Both daytime and nighttime noise was significantly higher in the diagnosed headache group than in the non-diagnosed group. Headache duration increased by daytime and nighttime noise with statistical significance in age-adjusted models.

**Conclusion:**

We found that noise exposure is correlated to headaches in children and adolescents. Daytime and nighttime environmental noise exposure was significantly associated with the duration of headaches through our data. Therefore, we assume that noise exposure is vitally relevant to prolonged headaches in the pediatric population. Further research is needed to improve our data.

## Introduction

Headaches are the most common neurologic symptoms in general populations, and approximately 90% of adolescents report episodes of head pain in their lifetime [1]. Most children suffer from headaches without serious underlying causes, but prolonged headaches induced to deteriorate the quality of personal daily life [2, 3]. Primary headache in children and adolescents focuses on associated factors such as related symptoms, family or medical history, and living environment factors [4] Among various related factors, auditory discomfort is related to recognizing the pain [5].

Noise-induced stress related to enhancing inflammatory pain in animal experiments [6]. In a human population study, headache patients who suffered regular attacks with exposure to noise stimuli reported higher rates of pain and lower tolerance to aversive stimuli [7]. Several previous studies revealed the association between environmental noise and headaches. Korean Working Condition Survey reported that occupational noise is related to adult headaches [8]. The German population-based study suggested the indirect linkage between residential noise and headaches. They reported that traffic noise-induced annoyance and insomnia were positively associated with adult headaches [9]. Noise stress-induced pain response in humans, particularly men, reacted with increased pain reactivity when exposed to a series of loud noise bursts [10].

Hypersensitivity to sound induce more frequent attack and a more potent pain scale in the population with migraine [11]. By self-reporting questionnaire study, patients with head pain showed a relatively lower threshold of sound discomfort but did not correlate to duration, frequency, or severity of pain [12]. In a long-term follow-up study, children and adolescents with migraine and tension-type headaches sustained pain with higher sensitivity to sound stimuli [13, 14]. However, noise stimulants are unclear to be defined as determinants of headaches in the pediatric population. We aimed to analyze the headache profile of pediatric populations and the connection between noise exposure and head pain in children and adolescents.

## Methods

### Study population and subgrouping

We retrospectively reviewed the medical records of patients complaining of headaches who visited Gyeongsang National University Changwon Hospital from January 2022 to April 2023.

**Fig. 1:**
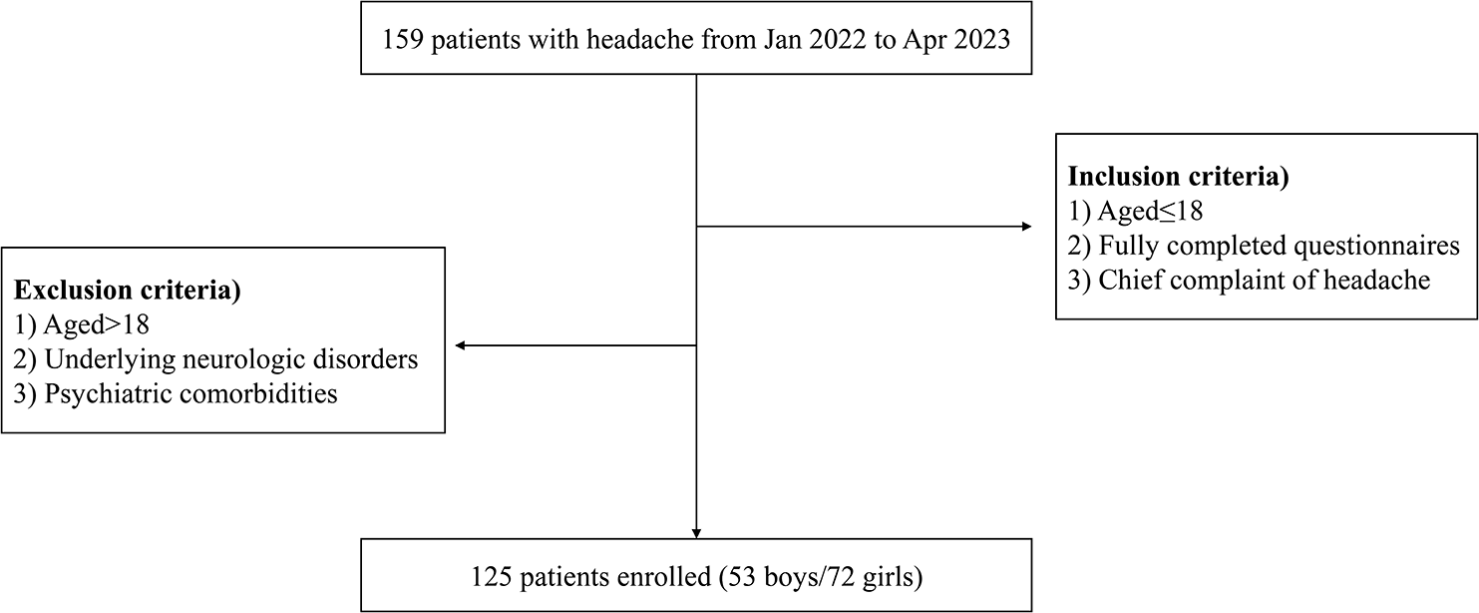
Flowchart of patient enrolment in this study

The inclusion criteria were as follows: (1) aged below 19 years, (2) fully completing the questionnaire with or without support from their parents, (3) mainly chief complaints of headache. A pediatric headache specialist confirmed the diagnosis of headache as a primary headache according to the International Classification of Headache Disorders, third edition [15]. A total of 224 participants enrolled, 125 subjects who suffered from headaches were finally included in the patient group **(**Fig. 1**)**.

The exclusion criteria were as follows: (1) aged above 19 years, (2) patients with structural brain abnormalities or underlying other neurologic conditions such as cortical deformities or malformations, traumatic brain injuries, cerebral palsy, encephalopathy, encephalitis, epilepsy, other genetic disorders, (3) patients with psychiatric comorbidities, including intellectual disabilities, attention deficit and compulsive disorder (ADHD), and mood disorders.

All methods followed relevant guidelines and regulations, and informed consent was obtained from all subjects and their legal guardians. This research was approved by the Institutional Review Board of Gyeongsang National University Changwon Hospital (IRB No: GNUCH 2023-03-044).

### Headaches

We analyzed the general characteristics of headaches based on medical records and headache diaries, including information on headache profiles such as duration (hours per day), frequency (times per week), characteristics of pain, associated factors (alleviating and/or triggering) and symptoms (nausea, vomiting, dizziness, blurred vision, abdominal pain), lifestyle factors (sleep cycle, physical activity, diet), and the response of painkiller. We used a widely used non-verbal measurement technique, the visual analogue scale (VAS), to evaluate the level of pain strength, 1 to 10 (1, none; 10, the most painful).

### Environmental noise

Environmental noise data from the National Noise Information System (NNIS), were used to analyze the relationship between noise exposure and headache. The NNIS offers two types of environmental noise data based on measurement methods: automatic and manual. In this study, the noise levels by district were identified using NNIS noise data, which were manually obtained. For the manual method, noise was measured at a height of 1.2–1.5 m from the ground and at locations without obstacles within a 3.5 m radius from the measurement spot. The measurement spots were selected to represent the regional noise level, avoiding locations close to specific noise sources, such as factories, construction sites, and airports. Environmental noise data can be categorized as either daytime or nighttime noise depending on the measurement time. Daytime noise was measured four times between 6:00 and 22:00, and nighttime noise was measured twice between 22:00 and 6:00, with an interval of at least two hours between measurements. Noise data with inaccurate measurement locations were removed from the NNIS dataset for preprocessing, and the preprocessed data differed in the number of measurement locations per year, ranging from a minimum of 1188 spots in 94 districts to a maximum of 1681 spots in 120 districts.

Because there were several administrative districts that did not involve measurement spots, and the spots represent a spatially uneven distribution, the noise levels of missing areas had to be estimated. In general, the spatial distribution of noise levels is primarily influenced by the locational characteristics of noise sources; thus, environmental noise levels can be regarded as a geographical phenomenon [16]. Therefore, it is feasible to estimate the noise levels of the entire target area through spatial interpolation. In this study, the environmental noise levels for all target districts were determined after interpolating the collected data by employing Empirical Bayesian Kriging (EBK), one of the kriging techniques. The EBK interpolates data spatially by iterating model improvement and value estimation. Unlike conventional kriging, EBK adopts a local model simulation. Furthermore, EBK is appropriate for estimating values in missing areas using a small amount of data. An environmental noise raster map for the entire area was created using EBK, based on the collected noise values, and the means of the noise values in the administrative district units were calculated.

### Covariates

Baseline characteristics, such as age, sex, and their habitat, were used in the current study. Children and adolescents were confirmed of their puberty status based on chronologic ages and self-assessments by questionnaire with or without the support of their parents [17, 18]. The questionnaires we used comprised various pubertal features based on the Pubertal Development Scale (PDS), Sexual Maturation Scale (SMS), and growth measurements cited by the World Health Organization [19–21].

### Statistical analysis

Statistical analyses were performed using SAS statistical software (version 9.4; SAS institute Inc., Cary, NC, USA). Chi-square tests were used to compare differences in base characteristics between cases and controls. Linear regression models were developed for daytime/nighttime environmental noise level and pain profile, including VAS, duration and frequency. For all statistical calculations, a two-tailed *P* value < 0.05 was considered statistically significant.

## Results

The general characteristics of the patients according to the presence of headaches are shown in Table 1. Of the 224 participants, 125 were clinically diagnosed with headaches, of whom 53 were boys and 72 were girls. Of the 104 pubertal subjects, 56.7% were diagnosed with headaches, compared to 60% in the prepubertal group. Both daytime and nighttime noise levels were significantly higher in the diagnosed headache group than in the non-diagnosed group.

**Table 1:** The basic and headache characteristics of study participants according to headache

	Headache, n (%) or mean (standard error)		*P*-value
	No	Yes	
Total participants	89 (41.6)	125 (58.4)	
Sex			0.8365
Boys	39 (42.4)	53 (57.6)	
Girls	50 (41.0)	72 (59.0)	
Subgroups			0.6285
puberty	45 (43.3)	59(56.7)	
prepuberty	44 (40.0)	66(60.0)	
Daytime noise (dB)	56.67 (0.27)	57.22 (0.19)	0.0378
Nighttime noise (dB)	48.53 (0.25)	49.04 (0.17)	0.0313

**Fig. 2:**
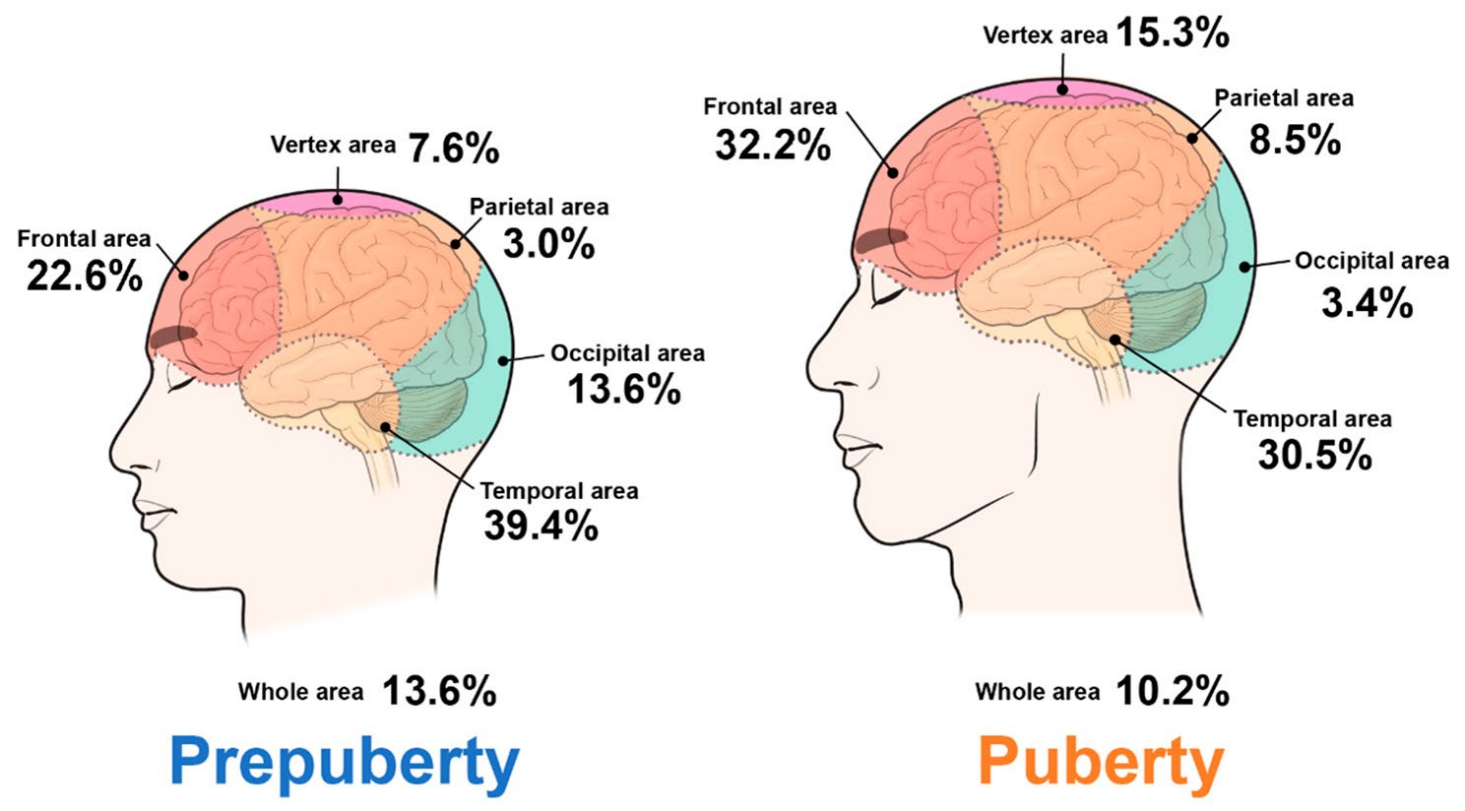
Distribution scheme of both subgroups with headache

Table 2 shows the characteristics of headaches in different subgroups. Boys were more likely to be diagnosed with headaches during puberty (54.2%), whereas girls were more likely to be diagnosed with headaches during prepuberty (68.2%). Age differences were also observed in headache types (migraine: girls 61.4%, boys 38.6%; and tension-type: girls 54.4% and boys 45.6%, *p*-value 0.4308). Further, a statistically higher proportion of patients experienced migraines in prepubertal and tension-type groups than in pubertal group. There were no significant differences in lateralization between each group, but frontal and temporal areas were the majority of headache area **(**Fig. 2**).**

**Table 2:** The headache characteristics with subgroups stratification

	Subgroups n(%) or mean(standard error)		*P*-value
	Puberty	Prepuberty	
Total	59 (47.2)	66 (52.8)	
Sex			0.0117
Boys	32 (54.2)	21 (31.8)	
Girls	27 (45.8)	45 (68.2)	
Headache type			< 0.0001
Migraine	14 (23.7)	43 (65.2)	
Tension-type	45 (76.3)	23 (34.9)	
Lateralization			0.1232
Unilateral	41 (69.5)	37 (56.1)	
Bilateral	18 (30.5)	29 (43.9)	
Family history of headache			0.1347
yes	25 (42.4)	25 (37.9)	
no	31 (52.5)	41 (62.1)	
N/A	3 (5.1)	0 (0.0)	
Response to painkiller			0.1750
yes	32 (54.2)	39 (59.1)	
no	24 (40.7)	27 (40.9)	
N/A	3 (5.1)	0 (0.0)	
Interference of daily activities			0.1373
Yes	28 (47.5)	31 (47.0)	
No	24 (40.7)	33 (50.0)	
N/A	7 (11.9)	2 (3.0)	
VAS^a^	6.51 (0.21)	6.45 (0.16)	0.0883
Duration^b^	11.32 (1.55)	10.06 (1.34)	0.4818
Frequency^c^	4.16 (0.37)	3.77 (0.30)	0.2517

^a^VAS, visual analogue scale (1, none; 10, most painful); ^b^Duration, prolonged hours per a day; ^c^Frequency, times per a week; N/A, not answered

Dizziness (54.9%) and nausea (32.8%) were prominent features of the associated symptoms, and the highest aggravating factor was studying (23.0%). Most patients felt that resting (40.2%) might help relieve pain, but a similar rate showed that none of the things (38.5%) helped alleviate their pain. Two-thirds of patients responded to painkillers, and the most common medicine was acetaminophen (45.1%). The headache score (VAS), duration, and frequency were all higher in the pubertal group than in the prepubertal group, but this difference was not statistically significant. Most participants in both subgroups consumed a regular diet, and no statistical correlation was established between any variables of the headache profile. The prepubertal group showed an average physical activity of 13.9 h per day, but the pubertal group showed an average of 2.6 h per day. A short period of sleep duration, defined as < 8 h per night [22–24], was significantly higher in the prepubertal population (57.8%, *p*-value < 0.0001) than in the pubertal group. However, no statistically significant correlation was observed between headache type and sleep duration in either subgroup.

The associations between daytime and nighttime noise exposure and headache characteristics are shown in Table 3. Among the three headache characteristics, duration was a more significant statistic than the other variables. In the age-adjusted models, headache duration increased by 0.8671 (± 0.2963) hours per day with each increase in 1 dB of daytime noise and by 0.9445 (± 0.3226) hours per day with each increase of 1 dB of nighttime noise with statistical significance.

**Table 3:** Linear regression models for headache characteristics related with noise level

	Noise, (standard error)	
	Day-time noise	Night-time noise
Crude model		
VAS	0.0858 (0.0996)	0.0955 (0.1084)
Duration	**0.8845 (0.2932)**	**0.9624 (0.3191)**
Frequency	0.1199 (0.0826)	0.1140 (0.0901)
Age-adjusted model		
VAS	0.0838 (0.1005)	0.0919 (0.1095)
Duration	**0.8671 (0.2963)**	**0.9445 (0.3226)**
Frequency	0.1333 (0.0830)	0.1276 (0.0905)

Bold indicated statistically significance.

## Discussion

Our results indicate that noise exposure is associated with pediatric headaches. Among the various precipitants of headaches, environmental noise affects children’s health conditions [25]. Based on previous environmental noise surveys, the WHO recommends not exceeding specific noise levels in children’s living areas [26, 27]. According to a study in Australia, noise is the most frequently endorsed precipitant in youth populations [28]. In a report by UAE, the second most common environmental exposure for school-aged children with migraines was loud noises [29]. A Swedish study performed that higher environmental noise was related to an increase in the prevalence of headaches in schoolchildren [30]. Thus, noise exposure is strongly related to headache provocation in the pediatric populations.

The majority of our patients, regardless of whether they were experiencing migraines or tension-type headaches (TTH), reported experiencing pain in the frontal or temporal areas. Our study established a significant statistical connection between daytime or nighttime noise and headaches. These findings led us to hypothesize that both the frontal and temporal cortices of the brain might play a crucial role in pain perception and could be physiologically linked to sustained pain in pediatric patients with headaches. The temporal cortex, responsible for auditory perception, and the frontal cortex, responsible for the processing and organizing perceptual information from sound, are key players in this process [31–33]. Building upon our results and aligning them with established views, our study suggests that exposure to sound stress can prolong the duration of head pain in children by affecting frontal or temporal perception.

Auditory stress may affect attention in school-aged populations. Based on our data, the pubertal group with headaches showed a slightly increased interference with daily activities compared with the prepubertal group with headaches **(**Table 2**)**. Auditory processing maturation occurs in at least 9-year-old children via the temporal cortex, and even mild auditory disorders can influence cognitive performance during adolescence [34, 35]. Therefore, we assume that predominantly distributed pain areas may interrupt the perceptual maturation processing of the brain in children and adolescents.

We found that noise exposure correlated with a longer duration of headaches in children. Environmental stimuli such as noise and light are related to the mechanism of increased sensitivity to migraine headaches [36]. Specifically, auditory stimuli induced the cochlear disturbance, which provoked the excitability of auditory neurons. It recruits the disruption of the normal inhibitory or facility of the higher neuronal center, including the brainstem. Thus, the mechanism of noise exposure could affect the hearing threshold and loudness sensitivity. Moreover, sound stress-induced hyperalgesia is an abnormally heightened sensitivity to pain. Khasa et al. previously reported enhanced levels of immune mediators related to hyperalgesia induced by unpredictable sound stimuli and prolonged, long-lasting pain in a rat model of widespread chronic pain [6, 37]. This indicates that auditory stress may be related to sensitivity and prolongation of pain in neuro-provoked mechanisms in pediatric headaches.

The connection between noise and headache may involve stress-related pathophysiological changes in the human body [5]. Most children are influenced by their daily behavior and performance owing to environmental stress. Noise exposure in children and adolescents is related to headaches and various psychosocial issues, including cognitive performance. In an Austrian survey, young children exposed to high-intensity noise showed increasing stressor indices, including systolic blood pressure, heart rate reactivity, cortisol level and decrements in mental health [38, 39]. In a previous cross-national study, noise exposure was associated with increased annoyance and impaired memory and reading comprehension in children [40]. Further, in a cross-sectional study in Macedonia, school children exposed to noise levels above WHO guidelines showed decreased attention and social adaptability and increased opposing behavior [41]. Chronic non-auditory noise can also affect intellectual performance in children [42]. Prolonged headaches in children and adolescents can induce poor cognitive performance and decrease the quality of daily life [43]. Therefore, environmental noise exposure should be considered a significant precipitant for managing pediatric health care.

This study has some limitations. First, we performed a retrospective study based on self-assessment. We analyzed the various factors related to headache profile, including associated symptoms or factors, although it is hardly excluded to designated factors because of data from self-questionnaires. We excluded sound or noise as a trigger or associated factors as much as possible. In the subgrouping, we assessed the stages of puberty based on questionnaires completed by the participants themselves or their parents. We did not collect serological markers such as serum hormone levels or cerebrovascular imaging. Additionally, we collected a relatively small population and a short enrollment period for exposure evaluation to compare other large-scale studies. We need to conduct more comprehensive follow-up studies on a larger scale in order to build upon these results.

Second, noise exposure levels can be biased by estimating residential addresses based on assumed indoor noise levels and outdoor measurements. However, previous studies have demonstrated a high correlation between indoor and outdoor noise levels [44, 45]. Although noise has been estimated using GIS spatial analysis for areas without noise information, limitations still exist. Noise measurement stations are designated to be representative of noise levels by considering factors such as population and area. In addition, the energy of noise decreases with distance from the measurement station, and the measurement or estimation may be less representative due to the influence of obstacles such as mountains or buildings.

Third, environmental characteristics such as light, air pollution, and ambient temperature of headache were not included in this study. Further studies should be conducted to confirm current results, including more detailed information on environmental characteristics as exposure factors. Nevertheless, this study has merit, as, to our acknowledgement, this study is the first report to reveal the correlation between environmental noise exposure and pediatric headache in South Korea.

## Conclusion

In conclusion, we found that daytime and nighttime environmental noise exposure was significantly associated with headache duration in pediatric population. Further research is required to strengthen the evidence supporting these results.

## Data Availability

The datasets generated and analyzed during the present study are available from the corresponding author upon reasonable request.

## Abbreviations

NNIS: National Noise Information System
ADHD: Attention Deficit /Hyperactivity Disorder
VAS: Visual Analogue Scale
EBK: Empirical Bayesian Kriging
PDS: Pubertal Development Scale
SMS: Sexual Maturation Scale
TTH: Tension-type Headaches
